# Evaluation of intraarterial and intravenous cisplatin chemotherapy in the treatment of metastatic osteosarcoma using an orthotopic xenograft mouse model

**DOI:** 10.1186/s13046-016-0392-1

**Published:** 2016-07-16

**Authors:** Bernhard Robl, Sander Martijn Botter, Giovanni Pellegrini, Olga Neklyudova, Bruno Fuchs

**Affiliations:** Laboratory for Orthopedic Research, Department of Orthopedics, Balgrist University Hospital, Forchstrasse 340, Zurich, 8008 Switzerland; Laboratory for Animal Model Pathology, Veterinary Pathology, Vetsuisse Faculty, Zurich, Switzerland

**Keywords:** Intraarterial, Cisplatin, Osteosarcoma, Intravenous, Metastasis

## Abstract

**Background:**

Osteosarcoma is the most common primary malignancy of bone. Its treatment relies on the administration of neoadjuvant and adjuvant chemotherapy combined with surgery. Alternative to common intravenous (i.v.) administration of chemotherapeutic drugs, clinical studies also evaluated the benefit of intraarterial (i.a.) administrations. However, conflicting results were obtained when both routes of administration of cisplatin (CDDP), a gold standard drug in osteosarcoma treatment, were compared. In order to overcome clinical confounding factors, we evaluated both routes of drug administration in a mouse model of experimental osteosarcoma.

**Methods:**

We directly compared i.v. versus i.a. drug infusions of cisplatin (CDDP), in an orthotopic xenograft mouse model of metastatic osteosarcoma. We performed tumor monitoring using caliper and micro computed tomography and measured tumor perfusion using laser speckle contrast imaging. Histopathological changes were evaluated using hematoxylin and eosin staining as well as immunohistochemistry (cleaved PARP-1, CD31, HIF-1α).

**Results:**

First, an effective concentration of 4 mg/kg i.a. CDDP was determined that significantly reduced primary tumor volume. We used this concentration of i.a. CDDP and compared it to infusions of i.v. CDDP. Systemic (i.v.) CDDP only showed minor suppression of tumor growth whereas local (i.a.) CDDP strongly inhibited tumor growth and destruction of cortical bone in the tumor-bearing hind limb. Inhibition of tumor growth was linked to a reduced blood perfusion and resulted in increased amounts of tumor necrosis after i.a. CDDP. After treatment with i.a. CDDP, remaining viable tumor tissue responded by increasing expression of HIF-1α. Side effects due to administration of CDDP were minor, showing no differences in kidney damage between i.v. and i.a. CDDP. However, increased epidermal apoptosis in the foot was an indirect marker for locally increased concentrations of CDDP.

**Conclusions:**

Our findings demonstrate the great potential of local administration of cytotoxic chemotherapeutics, such as CDDP. Consequently, we provide a preclinical basis for a renewed interest in the clinical use of i.a. chemotherapy in osteosarcoma therapy.

**Electronic supplementary material:**

The online version of this article (doi:10.1186/s13046-016-0392-1) contains supplementary material, which is available to authorized users.

## Background

Bone cancers are among the deadliest cancers in adolescents, with osteosarcoma as its most common representative [1, 2]. Subsequent to the introduction of chemotherapy in the early 1970s, 5-year survival rates of osteosarcoma patients with localized disease increased from 20 % to above 60 % [1, 3]. Standard of care for osteosarcoma patients currently includes systemic, intravenous (i.v.) administration of neoadjuvant chemotherapy, combined with surgical resection of the primary tumor, followed by adjuvant chemotherapy. However, 5-year survival rates plateaued at 60 % and survival rates of patients with metastatic disease remained unchanged at a low 20–30 % until today [3].

In osteosarcoma treatment, the use of neoadjuvant chemotherapy is considered valuable, yet without directly improving event-free survival compared to immediate surgery [4]. Especially pathologic analysis of the tumor response has important prognostic value. Good responders (i.e. 90 % or greater tumor necrosis) achieve up to two times better survival rates compared to patients with poor histologic responses [5–7]. In addition, neoadjuvant chemotherapy reduces tumor volumes prior to surgical resection, facilitating limb sparing procedures [8]. Therefore, neoadjuvant chemotherapy with highly cytotoxic drugs in osteosarcoma is commonly accepted as standard of care. One of the chemotherapeutics always included in today’s osteosarcoma treatment regimens is cisplatin (Cis-diamminedichloroplatinum, CDDP). However, its use is limited by systemic toxicities such as ototoxicity and nephrotoxicity [9, 10].

Therefore, local, yet controlled application of CDDP may be advantageous. One way to achieve this goal is by local drug administration via the tumor feeding artery. Such intraarterial (i.a.) drug administrations were already successfully performed in the 1980s. These studies confirmed a higher bioavailability of the drug after i.a. infusion of CDDP [11–13], explaining superior histological response rates in osteosarcoma [14–16]. For instance, Wilkins *et al.* achieved a good response in 87 % of the patients if patients with localized osteosarcomas were treated with i.a. CDDP and i.v. doxorubicin [14], compared to only 41 % in similar patient cohorts where i.v. CDDP and i.v. doxorubicin were used [17], and 71 % in case of a three/four-drug regimen comprising methotrexate [6, 17–21]. In addition to better response rates, studies from the St. Luke’s Medical Center achieved 10-year survival rates of between 82 and 93 % using i.a. CDDP [14, 22]. These survival rates compare favorably to other studies with maximum 10-year survival rates of, at best, 64 % with an i.v. two-drug regimen [17, 23] or up to 70 % with an i.v. three/four-drug regimen [3, 24]. Similarly, canine osteosarcoma patients showed superior responses when CDDP was infused via the tumor feeding artery compared to i.v. infusions [25].

Although these results demonstrate a clear added value of i.a. CDDP in osteosarcoma treatment, a clinical trial comparing both routes of CDDP administration was unable to show a benefit of i.a. chemotherapy [10]. This discrepancy might be explained by the design of the study, dose adaptations, administration of multiple drugs (standard of care) or its multi-institutional approach. In another study comparing i.a. versus i.v. CDDP, superior tumor responses with i.a. CDDP were only seen in the context of a three-drug regimen, and not as part of a four-drug regimen [20]. However, tumor response rates with the three-drug regimen comprising i.a. (77 %) were similar to the rates found with a four-drug regimen (81 %).

In summary, these studies demonstrate the difficulty of evaluating the “true” efficacy of i.a. CDDP due to confounding factors such as administration of different combinations of chemotherapeutics and the large difference in reported survival rates (between 50 % and 71% already for i.v. CDDP) per treatment center [6, 26]. In addition, tumor heterogeneity and side effect management make it difficult to reliably interpret the results of trials comparing both methods in a clinical setting. In this study, encouraged by the initial promising clinical benefits of i.a. chemotherapy, we investigated, under experimentally controlled conditions, the effects of local (i.a.) versus systemic (i.v.) CDDP in a preclinical mouse model of osteosarcoma.

## Methods

### Cell culture and transduction

Human OS 143B cells (CRL-8303) were obtained from American Type Culture Collection (ATCC, USA) and cultured in DMEM (4.5 g/L glucose)/HamF12 (1:1) medium (Invitrogen, USA) supplemented with 10 % heat-inactivated fetal calf serum at 37 °C in a humidified atmosphere containing 5 % CO_2_. Previously, 143B cells were transduced with the *LacZ* gene [27]. In this study, 143B/LacZ cells were additionally infected with retroviral particles containing the mCherry sequence integrated into a pQCIXH backbone, similar as described elsewhere [28]. The original mCherry-containing pcDNA3.1 plasmid was a kind gift from Prof. M. Rudin (Institute of Biomedical Engineering, University and ETH Zurich). After transduction, 143B cells were selected in tissue culture medium with 1200 μg/ml of G418 (Merck, Germany) and 400 μg/ml of hygromycin (Merck, Germany) to stably express LacZ and mCherry.

### Animal care

Female 8-week-old severe combined immunodeficiency mice (CB17/Icr-Prkdc scid/Crl; Charles River Laboratories, Germany) were maintained in enriched individually ventilated cages with light/dark-cycles of 12 h/12 h. After delivery, animals were kept for at least a week without any interventions. Food and water was provided to the mice ad libitum. Animal care and experimental procedures were in accordance with the institutional guidelines and approved by the Ethics Committee of the Veterinary Department, Canton of Zurich, Switzerland (License Number 64/2013).

### Orthotopic tumor induction in mice

143B cells were grown to subconfluence, detached with Trypsin/PBS/0.05 % EDTA, resuspended in PBS/0.05 % EDTA and kept on ice until injected. Before tumor cell injections (TCIs), mice were anesthetized using injection anesthesia. TCIs into left hind limbs were performed similar to as described elsewhere [29]. Briefly, holes were pre-drilled into the medullar cavity of left tibias using sterile needles, before 10^5^ 143B cells were injected. After TCIs, mice were monitored weekly for development of primary tumors (see below). Once mice started limping due to the tumor burden, 0.1 mg/kg of intraperitoneal (i.p.) Buprenorphine (Temgesic; Reckitt Benckiser, UK) was given twice daily. At the end of the study, mice were sacrificed and lung metastases were counted as described [28].

### Tumor monitoring

#### Primary tumor monitoring

After TCIs, mice were monitored weekly using caliper and fluorescence measurements, similar as described [28]. Once human 143B osteosarcoma cells established measureable primary tumors (unambigous mCherry signal and a volume greater than 25 mm^3^), drug treatment was started. mCherry tumor fluorescence was measured using an IVIS Lumina XR imaging system (Caliper Life Sciences, Inc., USA) and quantified with Living Image v3.1 software (Xenogen Corporation, USA).

#### Micro computed tomography

Micro computed tomography (microCT) using a SkyScan1176 microCT system (SkyScan/Bruker, Billerica, USA) equipped with a 0.5 mm aluminum filter was conducted to yield high-resolution tomographs of mouse hind limbs. Scans were obtained from each animal at the end of the study at a working source voltage of 50 kV and a source current of 500 μA yielding a final image pixel size of 17.7 μm. Frame averaging of three and exposure times of 210 ms per projection were set. Each shot required a source rotation step of 0.7° yielding scan times of approximately 8 min per mouse. Post-acquisition three-dimensional image reconstitution was done in NRecon software v1.6.9.18 (Skyscan/Bruker, USA). Reconstituted images were segmented and bone volumes were calculated using CTAn v1.13.11.0 (Skyscan/Bruker, USA). For calculation of bone and tumor volumes, the region between the distal end of the patella (“start of selection”) and the bifurcation of tibia and fibula (“end of selection”) was used. Bone volumes were calculated using the following formula: Δcortical bone volume = bone volume_tumor-limb_ - bone volume_healthy-limb_. Three-dimensional images of the mouse tibias were made in Ctvox v.2.7.0 (Skyscan/Bruker, USA).

### Drug infusions

After induction anesthesia with 5 % isoflurane (Forane; AbbVie, Inc., USA), anesthesia was maintained with 2 % isoflurane during drug infusions. Mice were kept warm on a heating mat throughout the procedure. Intravenous infusions were performed via the tail vein using a 30G needle attached to a polyethylene catheter (Portex; Smiths Medical, Inc., USA) under control of a syringe pump (Legato; WPI, Inc., USA). Intraarterial infusions were performed similarly as described [30]. Briefly, after revealing the femoral artery proximal to the intratibial tumor, the femoral nerve and the femoral vein were protected by inserting a nitrile strip. Subsequently, the femoral artery was cut and in-house-made, polyethylene catheters were inserted and manually held in place. Drug (2 or 4 mg/kg CDDP; Sandoz, Austria, in 0.9 % NaCl; B. Braun Medical, Inc., Germany, containing 0.8 % patent blue V; Guerbet, France) or vehicle (0.9 % NaCl containing 0.8 % patent blue V) alone were infused in a total volume of 350 μl within 2 min under control of a syringe pump (Legato; WPI, Inc., USA) for three times (every 72 h). All manipulations were performed under a stereo microscope (SZX 10; Olympus, Inc., Japan) placed in a sterile working environment. Success of the infusion was controlled through observing the distribution of the blue dye across the hind limb. After removal of the catheter, slight pressure was applied to the injection site in order to prevent bleeding and the site of surgery was flushed with 0.9 % NaCl. The wound was closed with non-degradable silk sutures (7–0 silk; B. Braun Medical, Inc.) in an intermittent pattern. Surgical procedures for an individual i.a. drug infusion took on average 52 min.

In total, two studies were performed: 1) a “dose establishment study” to identify an effective concentration of i.a. CDDP (N ≥ 4), and 2) a “comparison study” (4 mg/kg i.a. CDDP (*N* = 11) or i.a. vehicle (*N* = 6) versus 4 mg/kg i.v. CDDP (*N* = 6) or i.v. vehicle (*N* = 6)). Overall, i.a. infusions were tolerated well, nevertheless, one mouse treated with 2 mg/kg i.a. CDDP and two mice treated with 4 mg/kg i.a. CDDP were sacrificed prematurely during the “establishment study”, due to excessive (>15 %) body weight loss. Throughout the “comparison study”, one mouse of the i.v. vehicle-group had to be sacrificed due excessive body weight loss. Two mice from the group of i.v. CDDP dropped out, one during injection, another one was sacrificed due to excessive body weight loss. One mouse of the i.a. vehicle group died during the third surgery for unknown reasons. Only one mouse from the i.a. CDDP group dropped out of the study, after being found dead in the cage for unknown reasons. Drop outs were excluded from the analysis.

### Hind limb blood perfusion measurements

Laser speckle contrast imaging of the hind limbs of mice was conducted using a moorFLPI Full-Field Perfusion Imager (Moor instruments Ltd., UK) while mice were fixed in supine position. Imaging was done under low-light conditions on a heating pad set to 37 °C. Analysis of perfusion was done using the recorded flux (arbitrary units) images and the moorFLPI Review software v3.0 (Moor instruments Ltd.) by placing regions of interest (ROIs) where the primary tumor developed as well as in the corresponding region of the contralateral limb. Flux-ratios were calculated using the following formula: flux-ratio = flux_tumor_/flux_contralateral_ x 100 %.

### Histological and immunohistochemical analysis

Shortly after euthanasia, primary tumors were cut in equal parts, one snap frozen and the other part decalcified, paraffin-embedded and stained using routine methods. All slides were scanned using a digital slide scanner (NAnoZoomer-XR C12000, Hamamatsu Photonics K.K., Japan) and images were obtained using the corresponding NDP.view2 software. Quantitation of tumor necrosis was conducted using frozen and paraffin-embedded, hematoxylin and eosin (H&E)-stained sections of the tumors, assessing manually the proportion of necrotic tissues versus the total amount of tumor tissue available in the sections. Immunohistochemistry (IHC) was applied on frozen tumor sections to detect apoptotic cells (anti-cleaved PARP1 rabbit monoclonal antibody, #5625S, Cell Signaling Technology, Inc., USA; 1:50), HIF-1α (anti-HIF-1α rabbit polyclonal antibody, NB100479, Novus Biologicals, LLC; USA; 1:500), CD31 (anti-PECAM-1 rabbit polyclonal antibody, sc-1506-R, Santa Cruz Biotechnology, Inc., USA; 1:1000) and Von Willebrand Factor (anti-factor VIII-related antigen (FVIII-Rag) rabbit polyclonal antibody, A0082, Dako-Agilent Technologies, Denmark; 1:100). All immunohistochemical stains were performed using a Dako Autostainer (Dako-Agilent Technologies). A minimum of five high power fields (10X magnification in NDP.view2) or if less, the maximum available tissue area were used for analysis using ImageJ v1.47 (U. S. National Institutes of Health).

In the H&E-stained kidney sections, at least 300 proximal tubules from four randomly selected cortical regions were analyzed by a veterinary pathologist (GP) and a researcher (BR) in a blinded fashion. Tubules exhibiting degenerative changes of the lining epithelial cells such as pyknosis, fragmentation and absence of the nucleus and cytoplasmic hypereosinophilia were counted and normalized to the total number of healthy tubules using ImageJ v1.47 (U. S. National Institutes of Health, USA).

Apoptotic cells in the epidermis of tumor-bearing and tumor-free hind limbs were counted by a pathologist (GP) on the digital scans of the H&E-stained sections and expressed as average number of apoptotic cells per cm of skin (2 cm of epidermis evaluated in each limb). Apoptotic keratinocytes (AKs) exhibited a small, strongly basophilic, often fragmented nucleus and a round-up intensely eosinophilic cytoplasm. Apoptosis was confirmed using IHC for cleaved caspase-3 on paraffin-embedded sections (anti-cleaved caspase 3 rabbit monoclonal antibody, #9664, Cell Signaling Technology, Inc; 1:50).

### Statistical analysis

The results were given as mean ± standard error of the mean (SEM) unless otherwise stated. If Gaussian distributions were assumed, population means were compared with one-way ANOVA (for analysis of metastases, bone volume, necrosis, IHC stains) or repeated measures two-way ANOVA (for analysis of body weights, tumor volumes, blood perfusion) using Prism 5 v5.01 software (GraphPad Software, Inc., USA) followed by Bonferroni posttests. Using Prism 5, Pearson correlation calculations (HIF-1α versus CD31) as well as the Kruskal-Wallis test (tubular degeneration) and the Wilcoxon matched pairs test (number of AKs) were performed. Fisher’s exact test was calculated using SPSS Statistics v22 (IBM, USA). All statistical tests were 2-sided and *p* < 0.05 was regarded as statistically significant.

## Results

### Establishment of an i.a. drug injection model for treating osteosarcoma

**Fig. 1:**
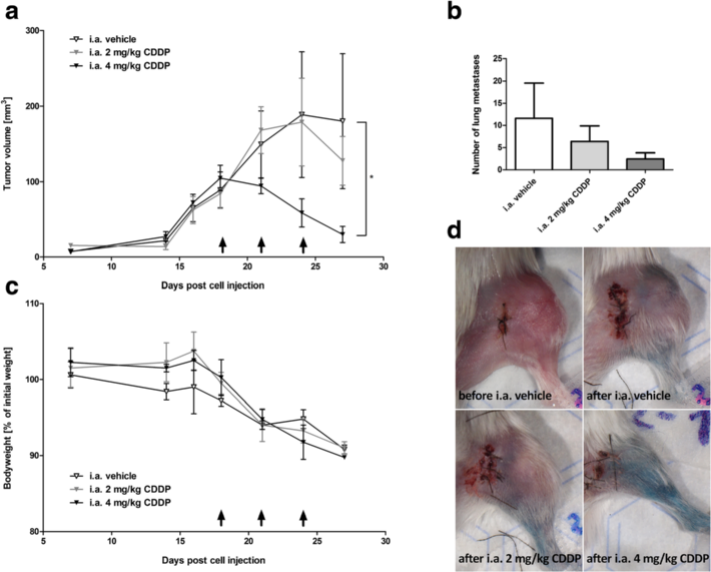
Identification of an effective concentration of i.a. CDDP. **a** Tumor volumes after three separate treatments with 0, 2, or 4 mg/kg of i.a. CDDP. Tumor volumes were determined by caliper measurements. **b** Presence of pulmonary metastases after treatment with 0, 2, or 4 mg/kg of i.a. CDDP. Metastases on the surfaces of lungs were counted ex vivo after X-gal staining. **c** Changes in body weight as an indicator for general health of the mice. **d** Examples of tumor-bearing hind limbs; before the third infusion of i.a. vehicle (upper left) and after the third successful infusion of i.a. vehicle (upper right), 2 mg/kg i.a. CDDP (lower left) and 4 mg/kg CDDP (lower right). The appearance of the blue color across the leg indicated a successful infusion. Days of drug infusion are indicated by black arrows (). *p < 0.05 as compared to the vehicle

First, the concentration of i.a. CDDP that led to a significant reduction in primary tumor growth was identified, which would later on be used in comparison with i.v. CDDP. After orthotopic injection of 143B osteosarcoma cells, different concentrations of CDDP in NaCl (0.9 %) vehicle were i.a. infused into the femoral artery of the tumor-bearing limb. Only 4 mg/kg (30 ± 11 mm^3^) and not 2 mg/kg (128 ± 30 mm^3^) of i.a. CDDP resulted in significant retardation of tumor growth compared to the vehicle (180 ± 89 mm^3^; Fig. 1a). Moreover, X-gal staining of tumor cells on the surface of lungs revealed a trend towards a dose-dependent reduction of lung metastases after i.a. CDDP (Fig. 1b). Administration of chemotherapeutics such as CDDP in preclinical models often leads to body weight loss but no significant differences between vehicle, 2 mg/kg i.a. CDDP and 4 mg/kg i.a. CDDP were noted (Fig. 1c). Every drug infusion was assessed visually by observing a color change from white to blue of the infused areas after successful infusion (Fig. 1d). Infusion quality controls indicated homogeneous dye distribution after three infusions of 4 mg/kg i.a. CDDP, whereas vehicle and 2 mg/kg i.a. CDDP yielded inhomogeneous dye distributions within the region of tumor growth.

### Osteosarcoma development dependent on the route of CDDP administration

**Fig. 2:**
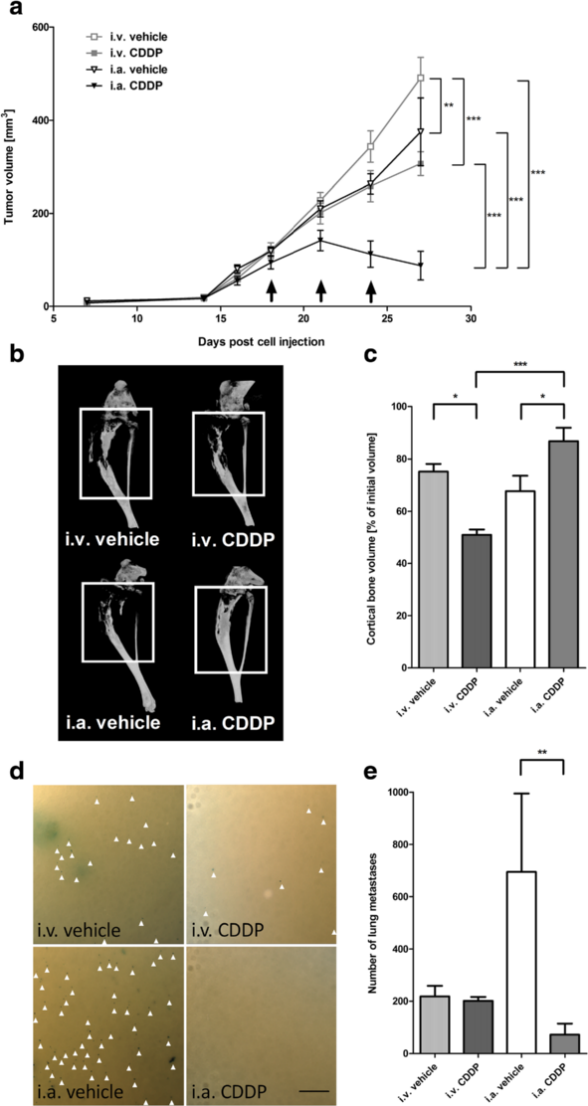
Effects of different routes of CDDP administration on osteosarcoma development. **a** Tumor volumes after treatment with vehicle or 4 mg/kg CDDP, both given i.v. and i.a.. Tumor volumes were determined by caliper measurements. Days of drug infusion are indicated by black arrows (). Two-way ANOVA: significant differences are only indicated for day 27. **b** Representative microCT scans of tumor-bearing bone. White squares mark the areas between the distal end of the patella and the bifurcation of tibia and fibula, which was used for quantification of differences in cortical bone. **c** Quantitation of differences in cortical (mineralized) bone volume as determined by microCT measurements. **d** Representative images of X-gal stained lung metastases. White arrowheads (∆) indicate lacZ^+^ lung metastases. Scale bar corresponds to 500 μm (4X). **e** Quantitation of number of pulmonary metastases on the entire lung surface. *p < 0.05; **p < 0.01; ***p < 0.001 as compared to the indicated treatment

Next, a comparison between i.v. and i.a. CDDP infusions was conducted. Only i.a. CDDP (88 ± 31 mm^3^) inhibited tumor growth and caused regression of primary tumors, while tumors continued to grow in all other treatment groups (tumor volumes at 27 days post tumor cell injection: i.v. CDDP: 307 ± 25 mm^3^; i.a. vehicle: 375 ± 73 mm^3^; i.v. vehicle: 491 ± 44 mm^3^; two-way ANOVA: *p* < 0.0001; Fig. 2a). One day prior to sacrifice, mice were subjected to microCT scans. Tumor volumes measured within the resulting tomographs confirmed caliper measurements and yielded significantly smaller final tumor volumes in the group receiving i.a. CDDP (54 ± 35 mm^3^) compared to volumes measured in other treatment groups (i.v. CDDP: 297 ± 29 mm^3^; i.a. vehicle: 286 ± 58 mm^3^; i.v. vehicle 479 ± 34 mm^3^; ANOVA: *p* < 0.0001). Osteosarcoma is known to be associated with pathological bone remodelling and increased fracture risk, and thus, the structural integrity of the bone influences the quality of life of osteosarcoma patients. 143B cell-derived osteosarcomas were shown to behave mostly osteolytic *in vivo* and loss of cortical bone correlates with increasing tumor volume. Accordingly, administration of i.a. CDDP (87 ± 5 % of initial bone volume (before treatment)) led to the smallest loss of cortical bone compared to i.v. vehicle (75 ± 3 %), i.a. vehicle (68 ± 6 %) and i.v. CDDP (51 ± 2 %; ANOVA: *p* < 0.0001; Fig. 2b, c).

Finally, X-gal staining of lacZ tagged cells on the surface of lungs collected during necropsy (Fig. 2d) showed that systemic i.v. CDDP had no significant effect on metastatic spread towards the lung compared to i.v. vehicle control (i.v. CDDP: 202 ± 15; i.v. vehicle: 218 ± 41). In contrast, i.a. CDDP significantly reduced the number of lung metastases (i.a CDDP: 82 ± 42; i.a vehicle: 695 ± 300; ANOVA: *p* < 0.001; Fig. 2e). Of note, a nonsignificant, but on average higher amount of metastases was found in i.a. vehicle versus i.v. vehicle group.

### Effect of CDDP treatment on tumor blood perfusion

**Fig. 3:**
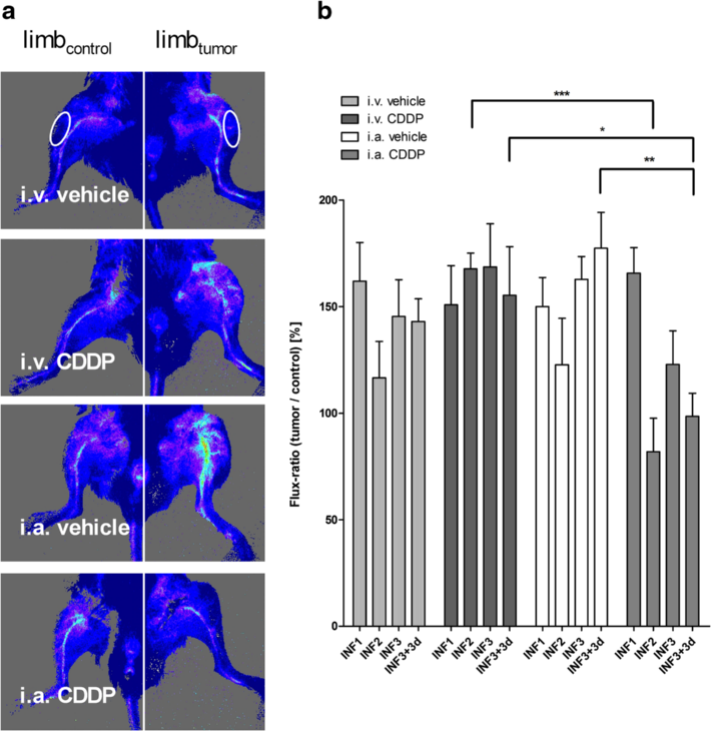
Changes in hind limb blood perfusion during the treatment period. **a** Representative images of perfusion measurements of the knee region from each treatment group at the end of the study (27 days post tumor cell injection). Images in the left column illustrate healthy contralateral limbs. Perfusion images in the right column illustrate tumor-bearing limbs. Circular ROIs (only indicated for “i.v. vehicle”) were used for measurements. **b** Flux-ratios of knee regions during the entire treatment period for individual treatment groups. Labeling of the x-axis indicates the respective measurement times: INF1/2/3: immediately prior to the first/s/third infusion; INF3 + 3d: three days after the final third infusion

Tumor-associated vasculature was assessed in vivo via blood perfusion measurements. Primary tumor growth induced an increase in perfusion of the tumor-bearing limbs compared to the contralateral control limbs (Fig. 3a). Following i.a. CDDP, a significant decrease in perfusion compared to i.v. CDDP or i.a. vehicle was observed (two-way ANOVA: *p* < 0.05; Fig. 3b). Interestingly, the largest reduction in perfusion were detected after i.a. CDDP infusions. At the end of the study, perfusion of the i.a. CDDP-treated limbs was close to physiological values, similar to the contralateral knee region. However, areas formerly infiltrated by osteosarcomas appeared poorly perfused, indicating the occurrence of ischemic tumor necrosis (e.g. Fig. 3a: i.a. CDDP).

### Histologic response to CDDP chemotherapy

**Fig. 4:**
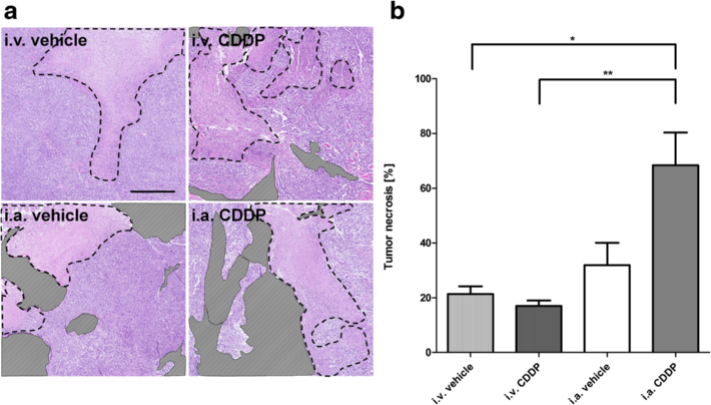
Histological evaluation of tumor necrosis. **a** Representative examples of necrotic (black dashed lines) tumor areas are shown for each treatment group. Areas overlaid with a striped pattern were not considered for evaluation (e.g. bone, muscle, absence of tissue). Scale bar corresponds to 500 μm (5X). **b** Quantitation of tumor necrosis, normalized to the total tumor area which was available for analysis. *p < 0.05 as compared to the indicated treatment

Assessment of tumor necrosis after neoadjuvant chemotherapy is an established endpoint to evaluate the response to treatment in osteosarcoma patients. Figure 4a illustrates representative examples from each treatment group which were used for the analysis of tumor necrosis. In case of two animals treated with i.a. CDDP, no tumor tissue could be found on cross sections of the tumor-bearing limb, indicating a strong anti-tumor effect (100 % of tumor necrosis was assumed). The largest mean tumor necrosis was detected after i.a. CDDP (68 ± 12 %) compared with i.a. vehicle (32 ± 8 %), i.v. CDDP (17 ± 2 %) or i.v. vehicle (21 ± 3 %, ANOVA: *p* < 0.01; Fig. 4b). According to Salzer-Kuntschik, a good responder is defined by more than 90 % tumor necrosis [31]. With i.a. CDDP, a total of five (45 %) good responses was achieved, whereas no good responses were detected with i.a. vehicle, i.v. vehicle or i.v. CDDP (Fisher’s exact test: *p* < 0.01; Additional file 1). Tumor cell death in the H&E-stained sections consisted of multifocal to coalescing, variably sized areas of necrosis: these areas which are, to varying extents, inherent to any rapidly growing tumor (i.e. after i.v. vehicle) are likely indicative of ischemic cell death.

### Influence of different routes of CDDP administration on remaining viable tumor tissue

**Fig. 5:**
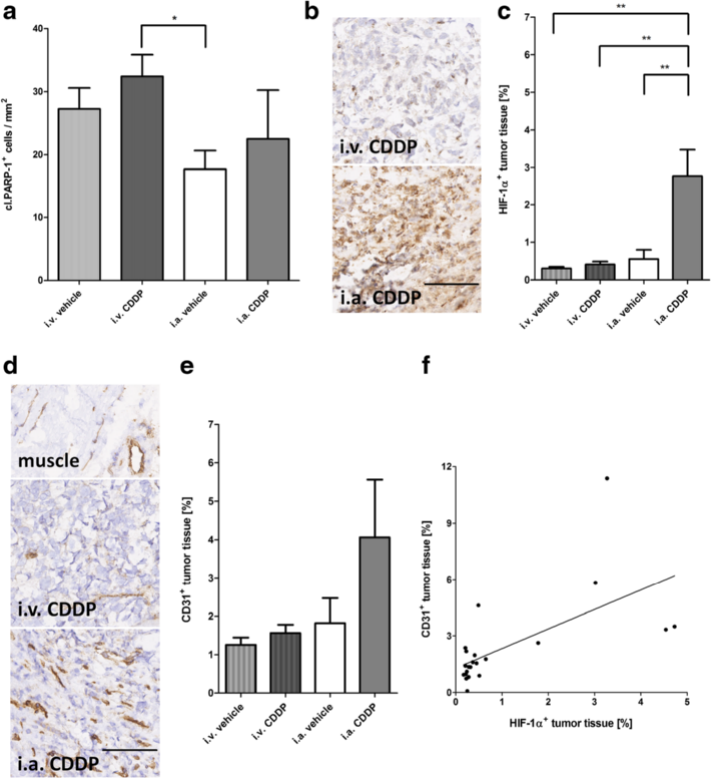
Effects of different routes of CDDP administration on remaining viable tumor tissue. **a** Number of cleaved PARP-1^+^ tumor cells. PARP-1^+^ tumor cells were only counted within areas of viable tumor tissue. **b** Representative images of tumor tissue (i.v. CDDP, i.a. CDDP) stained for HIF-1α (20X). Scale bar corresponds to 100 μm. **c** Quantitation of HIF-1α^+^ tumor tissue normalized to the entire viable tumor tissue available for evaluation. **d** Representative images of healthy adjacent tissue (muscle) and tumor tissue (i.v. CDDP, i.a. CDDP) stained for CD31 (20X), where the upper image shows CD31 expression in the endothelial cells lining capillaries as well as the larger vessels in the skeletal muscle surrounding the tumors, the central image represents CD31 expression after i.v. CDDP and the lower image displays an increase in CD31 expressing cells within the tumor mass after i.a. CDDP. Scale bar corresponds to 100 μm. **e** Quantitation of CD31^+^ tumor tissue normalized to the entire tumor tissue available for evaluation. **f** Correlation of HIF-1α IHC with CD31 IHC (Pearson’s r = 0.62). *p < 0.05; **p < 0.01 as compared to the indicated treatment

Due to the anti-tumor efficacy of i.a. CDDP, smaller areas of viable tumor were evaluated after treatment: i.a. CDDP (median 0.6 mm^2^; interquartile range 0.1–4.0 mm^2^) compared with i.a. vehicle (7.1 mm^2^; 3.9–11.3 mm^2^), i.v. CDDP (10.0 mm^2^; 7.2.–12.7 mm^2^) and i.v. vehicle (11.9 mm^2^; 11.0–14.8 mm^2^). Furthermore, two animals from the i.a. CDDP group were excluded from all immunohistochemical analyses involving viable primary tumor because of a total absence of tumor tissue. Within regions of viable tumor, scattered neoplastic cells exhibited morphological features of apoptosis, such as cell shrinkage, nuclear pyknosis and fragmentation, as indicated by immunohistochemical stains for cleaved PARP-1, a marker for chemotherapy-induced apoptosis [32]. However, no significant differences in the number of cleaved PARP-1^+^ cells within areas of remaining viable tumor were detected between corresponding vehicle and treatment groups (Fig. 5a).

In order to see if the reduced limb perfusion also resulted in increased hypoxia, expression of HIF-1α, a protein expressed under sub-physiological levels of oxygen, was studied. IHC of HIF-1α demonstrated intense staining of remaining viable tumor tissue after i.a. CDDP (Fig. 5b). Furthermore, quantitation of HIF1-1α expression demonstrated a significant increase in HIF-1α levels in tumors after administration of i.a. CDDP (2.8 ± 0.7 % of remaining viable tumor tissue) compared with tumors exposed to i.a. vehicle (0.6 ± 0.6 %), i.v. CDDP (0.4 ± 0.2 %) or i.v. vehicle (0.3 ± 0.1 %; ANOVA: *p* < 0.001; Fig. 5c). High levels of HIF-1α indicate low oxygen levels, subsequently triggering neovascularization. To this end, IHC for CD31 and factor VIII-related antigen (FVIII-RAg) was performed to characterize and quantify newly formed blood vessels within the neoplasms [33]. Examples of CD31 IHC are shown in Fig. 5d. In all groups, areas of viable tumor contained negligible numbers of FVIII-Rag^+^ blood vessels, while the endothelial cells lining large vessels in the skeletal muscle and connective tissue adjacent to the osteosarcomas expressed FVIII-RAg (data not shown). Quantitation of the CD31^+^ areas within viable tumor tissue indicated a trend towards increased neovascularization after i.a. CDDP (4.1 ± 1.5 % of remaining viable tumor tissue) compared with the lower levels observed after i.a. vehicle (1.8 ± 0.7 %), i.v. CDDP (1.6 ± 0.2 %) or i.v. vehicle treatment (1.3 ± 0.2 %; Fig. 5e). Furthermore, CD31 IHC significantly correlated with HIF-1α IHC (Pearson’s r: *p* < 0.01; r = 0.62; Fig. 5f).

### Side effects associated with different routes of CDDP administration

**Fig. 6:**
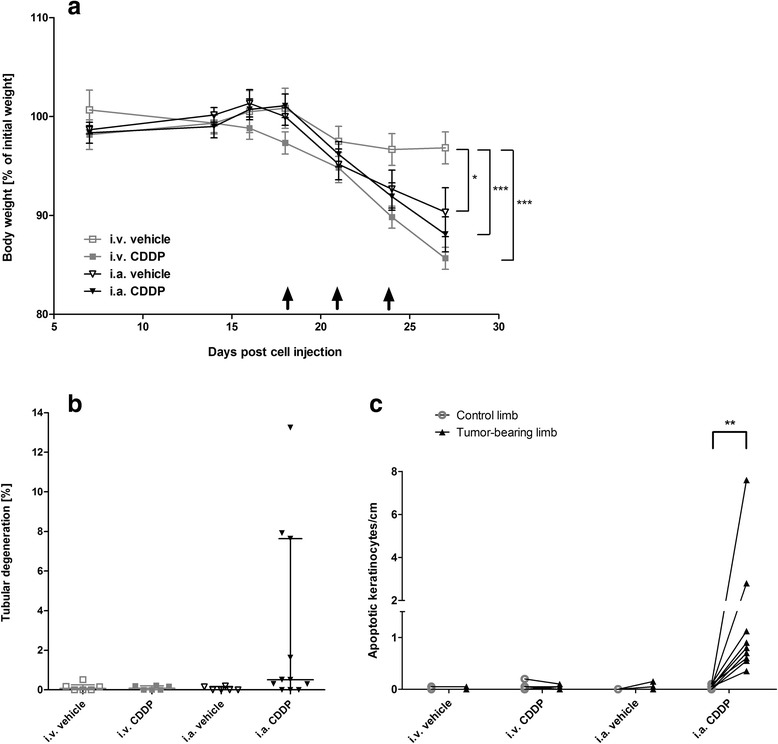
Effects of CDDP treatment on the health of the mice. **a** Monitoring of body weights during the study as an indicator for general health of the mice. Days of drug/vehicle infusion are indicated by black arrows (). Two-way ANOVA: significant differences are only indicated for day 27. **b** Quantitation of tubular degeneration at the end of the study. Tubular degeneration is displayed as the median ± the interquartile range. **c** Apoptotic keratinocyte counts. The number of apoptotic cells per cm of epidermis of the hind limbs was determined. The number of apoptotic keratinocytes was determined in healthy limbs (control limb) as well as tumor-bearing limbs of the same animal. Data points from the same animal are connected. *p < 0.05; ***p < 0.001

Similar to the “dose establishment study”, body weights were measured at regular intervals throughout the “comparison study” (Fig 6a). When comparing CDDP administrations only (i.a. CDDP: 88 ± 2 % of body weight normalized to the weight at day of tumor cell injection versus i.v. CDDP: 85 ± 1 %), no significant difference in body weight development between i.a. or i.v. CDDP administration was demonstrated. In contrast, i.v. CDDP caused a significant drop in body weight compared with i.v. vehicle (97 ± 2 %), whereas no difference was found between i.a. CDDP and i.a. vehicle (90 ± 2 %; two-way ANOVA: *p* < 0.0001; Fig. 6a).

In general, application of CDDP is limited by a high incidence of severe nephrotoxicity characterized by degeneration or death of the proximal tubule epithelial cells [34, 35]. In this study, however, no histological abnormality was recognized in the kidneys, except in three mice after i.a. CDDP, which exhibited mild acute tubular degeneration/necrosis, affecting only a small proportion of the renal tubules (Fig. 6b).

To assess whether i.a. CDDP resulted in higher apoptotic rates of non-malignant cells, suggestive of higher local concentrations of CDDP in the operated limb, the number of apoptotic keratinocytes within the skin of treated limbs and contralateral limbs were quantified. Results demonstrated lower numbers of apoptotic keratinocytes (AK) after i.v. vehicle (0–0.05 AK/cm of skin; minimum - maximum of tumor bearing limb), i.v. CDDP (0–0.1 AK/cm) or i.a. vehicle treatment (0–0.05 AK/cm), in tumor-bearing limbs as well as in the corresponding contralateral limb (Fig. 6c). In contrast, significantly higher numbers of AKs were found after the administration of i.a. CDDP (0.35–7.6 AK/cm), yet in the tumor-bearing limb only (Wilcoxon matched pair test: *p* < 0.01; Fig. 6c).

## Discussion

In this study, we present the successful establishment of i.a. drug administrations in a mouse model of experimental orthotopic osteosarcoma. Using CDDP as a gold standard drug for osteosarcoma treatment, we showed that our setup of i.a. drug infusions is feasible and that primary and systemic disease could be inhibited in a concentration-dependent manner. Moreover, we demonstrated that i.a. CDDP is more effective in inhibiting osteosarcoma progression than equivalent concentrations of i.v. CDDP, as indicated by smaller primary tumor volumes, decreased destruction of cortical bone as well as decreased numbers of lung metastases. Increased anti-tumor efficacy of i.a. CDDP infusions was also confirmed by histological analyses, where we demonstrated increased levels of tumor necrosis. Decreased tumor blood perfusion and increased hypoxia of the neoplasms after i.a. CDDP administration was demonstrated and explains, at least partially, the superior efficacy of localized CDDP delivery. Finally, we showed that i.a. CDDP causes increased levels of apoptotic keratinocytes in the epidermis of tumor-bearing limbs, while other systemic side effects were similar compared with i.v. CDDP.

Despite the use of larger animal model systems such as dogs [25] or sheep [36], the most commonly used model systems in osteosarcoma research are rodents [37]. To our knowledge, this is the first report of an i.a. infusion model in experimental, orthotopic osteosarcoma in mice, where we could demonstrate superior tumor control as compared to routine i.v. infusions. Hence, our i.a. model can be used as a platform for the investigation of other small molecules whose systemic application is limited by side effects. Especially for osteosarcomas of the limb, easy access to the tumor feeding artery offers a valuable alternative to systemic CDDP infusions. However, i.a. infusions are not limited to tumors of the limb. Another recent study using a mouse model of metastatic brain tumors demonstrated advantages in tumor control with i.a. chemotherapy (internal carotid artery) compared with i.v. chemotherapy (tail vein), similar to our study [38]. This demonstrates that even difficult-to-access tumor entities can be treated with i.a. drug infusions.

Local application of small molecules such as CDDP offers several advantages compared with systemic application. First, higher tumor drug loads can be achieved after infusion of equivalent drug concentrations via the tumor-feeding artery [11], causing greater tumor necrosis [13]. In line with this assumption, we demonstrated greater tumor necrosis after i.a. CDDP compared with i.v. CDDP administration. In contrast to Winkler et al., we were able to compare i.a. versus i.v. routes of administration in the absence of clinical confounding factors such as changes in drug infusion times or stratification of patients (e.g. high-risk only) [10]. In our study, i.a. CDDP not only caused a reduction of the primary tumor volume but also minimized the loss of mineralized bone- an indicator for experimental osteosarcoma progression [39]. In contrast, loss of cortical bone was increased after i.v. CDDP treatment compared with i.v. vehicle. Likewise, when sites of bone turnover in dogs were studied, bone remodeling was significantly influenced by the systemic administration of CDDP [40].

Systemic side effects, especially nephrotoxicity, are equal or reduced after local CDDP infusion compared to systemic application without a simultaneous reduction of the systemic potency of the drug [9, 20]. In our study, most animals showed no signs of nephrotoxicity. However, we found evidence of mild nephrotoxicity, represented by scattered tubular degeneration/necrosis in three animals treated with i.a. CDDP. Renal injury was minor and unlikely to have an impact on kidney function. It is possible that hypovolemia, resulting from blood loss and/or insufficient rehydration after the repeated surgical interventions, exacerbated the observed nephrotoxicity in these animals.

Skin necrosis is another side effect observed in human patients after i.a. administration of CDDP [10, 20]. However, this usually does not lead to complications during the treatment and regenerates well. Likewise, our results indicated that i.a. CDDP led to increased numbers of apoptotic keratinocytes in the tumor-bearing limbs, but not in the healthy contralateral limbs. Elevated numbers of AK may indicate a higher local CDDP concentration in the proximity of the primary tumor and thus, help to explain the superior response after administration of i.a. CDDP compared with i.v. CDDP. Interestingly, some mild chemotherapy-induced toxicities were shown to be associated with improved osteosarcoma patient survival [23].

Consistent with the observations reported by Wilkins et al., where a reduction of the spongy tumor vasculature after i.a. CDDP was detected [14, 22], we also observed a general decrease in perfusion of the tumor region shortly after i.a. CDDP administration. This reduction in tumor perfusion in addition to the higher local CDDP concentration may have further contributed to the regression of the experimental tumors and resulted in a good histological response (as defined by having at least 90 % tumor necrosis) in at least 45 % of the mice. Thus, destruction of the tumor vasculature seems to be a necessity for i.a. CDDP to successfully induce tumor necrosis potentially resulting in a high percentage of good histologic responses [14].

The reduction in tumor perfusion after i.a. CDDP might have caused the remaining viable tumor tissue to react by expressing increased levels of HIF-1α. It is known that constitutively active HIF-1α induces neovascularization and increased expression of CD31 or VEGF [41–44]. Increased expression of HIF-1α in osteosarcomas after i.a. CDDP was paralleled by increased microvascular density assessed using IHC for CD31, a marker of immature endothelium. In addition to CD31, consecutive tissue sections were stained for FVIII-RAg, normally found in large, mature vessels [33]. The few scattered FVIII-Rag^+^ vascular structures found within the tumors were likely pre-existing and no difference was found among the different groups. In summary, our results indicate a response of the neoplasms towards ischemic damage after i.a. CDDP by increasing HIF-1α-levels and potentially initiating neovascularization.

Physical manipulation of the primary tumor as well as changes of blood perfusion within the primary tumor is known to increase numbers of circulating tumor cells and thus, the risk for the development of metastases [45, 46]. In our study, this might be reflected by a higher, albeit nonsignificant amount of lung metastases after i.a. vehicle administration, which, following i.a. CDDP, was reduced below amounts following i.v. CDDP. Thus, in addition to improved local tumor control, i.a. CDDP also successfully controlled the number of spontaneous lung metastases. This is especially relevant in osteosarcoma, where controlling pulmonary metastases strongly influences patient survival [47–49].

One limitation of our study is the inherent variability due to any surgical procedure. Although the same surgeon performed i.a. drug infusions, the parameters of i.a. infusions varied (e.g. duration of surgery or degree of blood loss). Especially the placement of the catheter is critical for a homogeneous drug distribution in subsequent arterial branches [50] and thus, impacts success of therapy. In general, our study suggests a superior outcome in the chemotherapeutic response after i.a. delivery of CDDP, however, the individual outcomes must be interpreted alongside corresponding toxicokinetic information.

## Conclusions

Taken together, our study demonstrates the potential of i.a. CDDP in a clinically relevant osteosarcoma model. The superior primary tumor control of i.a. CDDP in our study demonstrates the potential of i.a. drug administrations as currently used in some clinics. Despite the greater technical requirements for i.a. drug infusions, we suggest that the potential of i.a. infusions in osteosarcoma treatment should be considered when evaluating (novel) compounds and combinations thereof. Especially for a rare disease such as osteosarcoma, we believe that our intraarterial therapy model can aid in the preclinical assessment of drug efficacy and thus, improve osteosarcoma patient treatment.

## Abbreviations

AK, apoptotic keratinocyte; CD31, cluster of differentiation 31; CDDP, cisplatin; FVIII-RAg, factor VIII related antigen; H&E, hematoxylin and eosin; HIF-1α, hypoxia inducible factor-1α; i.a, intraarterial; i.p, intraperitoneal; i.v, intravenous; IHC, immunohistochemistry; microCT, micro computed tomography; PARP-1, poly ADP ribose polymerase-1; SCID, severe combined immunodeficiency; TCIs: tumor cell injections

## Additional file

Additional file 1: Histological response according to treatment. Groups the histological tumor response according to current clinical criteria for evaluation of tumor necrosis. (DOCX 14 kb)
