# Supplementary material for: Evaluation of intraarterial and intravenous cisplatin chemotherapy in the treatment of metastatic osteosarcoma using an orthotopic xenograft mouse model
Source: J Exp Clin Cancer Res. 2016 Jul 16;35:113. doi: 10.1186/s13046-016-0392-1 (PMC4947253; doi:10.1186/s13046-016-0392-1)
Supplement: Additional file 1: — Histological response according to treatment. Groups the histological tumor response according to current clinical criteria for evaluation of tumor necrosis. (DOCX 14 kb) [file 13046_2016_392_MOESM1_ESM.docx]

| **Additional file 1** Histological response according to treatment | | | | | |
| --- | --- | --- | --- | --- | --- |
| Treatment | | Number | >90% tumor necrosis | 50 - 90% tumor necrosis | < 50% tumor necrosis |
|  | i.a. CDDP | 11 | 5 | 3 | 3 |
|  | i.a. vehicle | 6 | 0 | 2 | 4 |
|  | i.v. CDDP | 6 | 0 | 0 | 6 |
|  | i.v. vehicle | 6 | 0 | 0 | 6 |
| Fisher's Exact Test: | | *p* = 0.006 |  |  |  |
